# Risk factors for mild cognitive impairment among older adults in a hospital in southern Nigeria

**DOI:** 10.4102/phcfm.v15i1.3942

**Published:** 2023-04-25

**Authors:** Amaefuna C. Anieto, Akinwumi O. Owolabi, Mojisola O. Owolabi, Anthony I. Nwajei, Mabel O. Onwuka

**Affiliations:** 1 Department of Family Medicine, Federal Medical Centre, Asaba, Nigeria

**Keywords:** mild cognitive impairment, dementia, risk factors, prevalence, older adults, geriatric clinic

## Abstract

**Background:**

About 63% of people living with mild cognitive impairment (MCI) and dementia live in low- and middle-income countries (LMICs). Emerging evidence suggests that early risk factors for the development of MCI and dementia can be modified by public health and preventive intervention approaches.

**Aim:**

This study aimed to assess the prevalence of MCI in older adult patients and its relationship with some risk factors.

**Setting:**

The study was conducted among older adults at the Geriatric Clinic of the Family Medicine Department of a hospital in southern Nigeria.

**Methods:**

A cross-sectional study was carried out involving 160 subjects aged 65 years and above over a period of 3 months. Socio-demographic and clinical data were obtained using an interviewer-administered questionnaire. Subjects were accessed for impaired cognition using the 10-word delay recall test scale. Data were analysed using SPSS version 23.

**Results:**

There were 64 males and 96 females; male to female ratio was 1:1.5. Majority of the study population were in age range of 65–74 years. The overall prevalence of MCI was 59.4%. Respondents with tertiary education were 82% less likely to have MCI on logistic regression analysis (OR = 0.18, 95% CI = 0.465–0.719).

**Conclusion:**

Mild cognitive impairment was prevalent among older adults in this study and was found to be significantly associated with low level of education.

**Contribution:**

It is therefore recommended that screening for MCI and known risk factors should be prioritized at geriatric clinics.

## Introduction

Dementia and mild cognitive impairment (MCI) are major neurocognitive disorders that pose remarkable health challenges worldwide. Dementia can be quite dehumanising in its advanced stages. Mild cognitive impairment occupies the intermediate stages in the continuum of cognition and is considered the leading point for preventive strategies.^1^ Mild cognitive impairment is a condition that was first proposed by Petersen as a nosological entity that refers to older individuals with mild cognitive deficit without dementia;^2^ hence, it was initially termed Cognitive Impairment No Dementia (CIND).^2,3^ Subjects with MCI have mild but measurable changes in thinking abilities that are noticeable to the individual, family members and friends.^4^

Mild cognitive impairment was initially described as a single syndrome in which the individual displays subjective memory complaints and objective deficits in episodic memory tests, with no impairment in activities of daily living (ADL) and no dementia.^5^ Mild cognitive impairment differs from dementia because it is not severe enough to interfere with independence in daily life.^6^ Occasionally, clinical criteria dictate that the onset of dementia is preceded by a prodromal state of mild cognitive decline and individuals with MCI have been found to be at increased risk of dementia.^7^

The global prevalence of dementia is estimated at 5% to 7% in people over 60 years.^8^ Documented country-specific dementia prevalence in sub-Saharan Africa (SSA) ranges between 2% and 5%.^9^ The prevalence estimates were 18.4% and 2.9% for MCI and dementia, respectively, in a study carried out by Ogunniyi et al. in South-West Nigeria.^1^ A prevalence of MCI of 11.8% has also been reported from South-East Nigeria.^10^

The risk factors for MCI include increasing age and other medical conditions and lifestyle factors such as, diabetes, smoking, high blood pressure (BP), elevated cholesterol, obesity, depression, a lack of physical exercise, low education level and infrequent participation in mentally or socially stimulating activities.^11^ As a nosological entity, MCI conveys important health implications, in particular an increased risk of developing dementia in the near future.^12^ This is evident from studies of MCI patients presenting to the clinics with a memory disorder, in whom the annual rate of progression to dementia was reported to be between 10% and 15%.^12^ Dementia disrupts the normal functioning of affected individuals and their families, imposing significant social and economic burdens.^13^ This is especially severe in low- and middle-income countries (LMICs), where dementia is the most important independent contributor to disability in older adults, and resources to diagnose and treat dementia are limited.^13^ There is a paucity of information on major neurocognitive disorders in SSA where the number of individuals with neurocognitive disorders and other disease burden is expected to increase because of demographic transition.^1^

There is a need to assess the modifiable risk factors for MCI as this will help in determination of the appropriate interventions that are implementable to prevent or reduce the rate of cognitive decline among this group of people. Knowing the prevalence of MCI is also very important to create awareness and this will hopefully lead to early detection of MCI and dementia among older persons.

The aim of the study was to assess some of the risk factors for MCI in older adults aged 65 years and above in a tertiary care hospital in Southern Nigeria with the ultimate goal of reducing the burden of the condition. The specific objectives were to measure the prevalence of MCI and assess the relationship between sociodemographic factors and MCI. The study also aimed to assess the relationship between some risk factors for MCI such as obesity, alcohol use, tobacco smoking and hypertension, and MCI among this category of people.

## Research methods and design

### Study design and setting

The study was a cross-sectional study conducted in the Geriatric Clinic of the Federal Medical Centre, Asaba, Delta state in Southern Nigeria.

### Study population

These were older adults who are 65 years and above. An average of three participants were recruited per day from the Geriatric clinic, which runs 5 days a week. Data were collected over a period of 3 months.

### Inclusion criteria

All older adults who were 65 years and above were presented to the geriatric clinic.

### Exclusion criteria

Older adults with dementia, those who were too sick to participate in the study, those who were confused and had reduced awareness of their surroundings, and those who were grieving or diagnosed with psychiatric illnesses.

### Sample size estimation and sampling strategy

The sample size was calculated using the prevalence of cognitive impairment (11.8%) reported in a study performed in Dunukofia Local Government Area of Anambra state, Nigeria by Uwakwe et al., published in a World Health Organization (WHO) bulletin.^10^

Sample size was calculated to be 159.9 (160) using Leslie Kish formula for population > 10 000.^14^


$$n=\frac{z^{2}\text{pq}}{d^{2}}$$ [Eqn 1]

where:

*n* = sample size,

*z* = standard deviation of 1.96 at a confidence interval of 95%,

*p* = the proportion in the target population estimated to be affected (11.8%),

*q* = 1 – *p* and

*d* = desired degree of precision or accuracy = 5% (standard value of 0.05).

An average of 12 patients were seen daily in the Geriatric Clinic, which ran from Monday to Friday; this gave an estimate of 60 patients per week and 240 patients per month and 720 patients in 3 months.

Systematic random sampling technique was used utilising a sampling interval of five. The first participant was selected by balloting among the first five patients who met the inclusion criteria. Every 5th patient who met the inclusion criteria was recruited until the required sample size was reached. Any patient who did not meet the inclusion criteria was replaced with the next person and thereafter every 5th person. To avoid double enrolment, the folders of those enrolled were tagged with green stickers.

### Data collection

Katz Index^15^ was used in measuring the basic ADL. A mental state examination was also performed and in conjunction with katz index, were used to assess for and exclude subjects with dementia.^16,17,18^ Detailed medical history as well as mental state examination were also used to exclude subjects with confusion or delirium based on clinical assessment.^18^ Participants’ past medical histories were obtained by self-report and from hospital records. Data were collected using an interviewer-administered questionnaire. The questionnaire was used to collect information on age, gender, educational status, marital status, residence (urban or rural), religion and occupation of the respondents, as well as their self-reported tobacco smoking and alcohol use. Information on the history and duration of hypertension was obtained, BP measurement was performed, measurement of weight and height was carried out and body mass index (BMI) was calculated.

An adult weighing scale with a stadiometer was used to get the height and weight of the participants. The participants’ height measurement was recorded to the nearest 0.1 cm. The weight measurement was recorded to the nearest 0.1 kg. Body mass index was calculated with the formula: BMI = Weight (in kilograms) divided by height squared (in meters) and graded as follows: Underweight = BMI < 18.5, Normal = BMI 18.5 – 24.9, Overweight = BMI 25–29.9 and Obese = BMI ≥ 30.0.

Blood pressure was measured twice on the left arm using a mercury sphygmomanometer. The systolic was taken at the first Korotkoff sound and the diastolic was taken at the fifth Korotkoff sound. The mean reading of the BP was calculated. Using the JNC-8 criteria for age, a BP of ≤ 150 mmHg for systolic and ≤ 90 mmHg for diastolic was defined as normal while a systolic BP of greater than 150 mmHg and a diastolic of greater than 90 mmHg was defined as elevated.^19^

Mild cognitive impairment was assessed using the 10-word delay recall test (10-WDRT).^20^ The 10-word list adapted from the Consortium to Establish a Registry of Alzheimer’s Disease (CERAD) 10-word learning list, has been shown to be one of the more sensitive tests for detecting MCI.^20^ The 10-WDRT has been shown to be largely unaffected by educational level for assessment of dementia in Nigeria^21^ and has been shown to be reliable and valid with a validity score of seven out of seven.^10,21^ The 10-WDRT has two components: the immediate and the delayed recall. Immediate recall is a test of working memory. This consisted of initial memory trials using a 10-word list. Subjects were shown the words on the list. The words were read at a rate of two seconds. The subject repeated the same words and was prompted when necessary. Words correctly recalled were ticked but no points were given. The order of the recalled words did not matter. The second trial was performed following the first trial. The words were rearranged and read to the subject at the rate of every two seconds. The subject was asked to repeat the words and the correctly recalled words were ticked but no points were given. The words were rearranged again for the third trial and read at the rate of every two seconds. The correctly recalled words were ticked and no points were given.

The delayed recall is a test of judgement. The subject waited for 5 min and was then asked to recall the 10 words told to him or her earlier but this time without further cues. A point was given for each correctly remembered word. The maximum score was 10 out of 10 while the minimum score was zero. A diagnosis of MCI is made on a score of ≤ 2 in the 10-WDRT.

### Data analysis

The data were cleaned and coded then entered into SPSS version 23 software for Windows (IBM Corp. Armonk, NY, USA), which was used for the data analysis. Categorical variables were presented as frequencies and percentages while continuous variables were expressed as means and standard deviation. Univariate analysis was carried out to find prevalence while bivariate analysis was used to examine the relationships between respondents’ sociodemographic factors and risk factors such as obesity, alcohol use, tobacco smoking and hypertension and MCI. Chi square (χ^2^) test was used to test the difference in proportions for categorical variables. Logistic regression models were used to find significant predictors of MCI. A *p*-value of ≤ 0.05 was considered statistically significant.

### Ethical consideration

Permission and approval to carry out the study was obtained from the Research and Ethics Committee of the Federal Medical Centre, Asaba, Delta State, Nigeria (No. FMC/ASB/A81 VOL. XII/90). Written informed consent was obtained from all willing subjects after a full explanation of nature, benefits and possible discomfort that the subject may experience in taking their measurements and time that would be spent in the course of the interview before enrolment into the study. Necessary steps were taken to preserve patient anonymity and confidentiality. Willingness or unwillingness to participate in the study in no way interfered with the patients’ management.

## Results

A total of 160 respondents were enrolled for the study. There were more female respondents 96 (60%) with male to female ratio of 1:1.5. Respondents aged 65–74 years accounted for 98 (61.2%) of the participants. The majority of the respondents were retired (73.8%), about a third reside in urban areas (37.5%), a high proportion had primary school education (44.4%), a little over half were married (54.4%) and all the respondents were Christians (Table 1). The mean age was 72.9 ± 6.4 years and the age range was 65–92 years.

**TABLE 1:** Sociodemographic characteristics of the respondents.

Variables	Frequency *N* = 160	Percentage
**Gender**		
Female	96	60.0
Male	64	40.0
**Age (years)**		
65–74	98	61.2
75–84	52	32.5
> 85	10	6.3
**Occupation**		
Active	42	26.2
Retired	118	73.8
**Residence**		
Urban	60	37.5
Semi-urban	42	26.3
Rural	58	36.2
**Educational qualification**		
No formal	22	13.7
Primary	71	44.4
Secondary	32	20.0
Tertiary	35	21.9
**Marital status**		
Widowed	73	45.6
Married	87	54.4
**Religion**		
Christianity	160	100.0

Most of the respondents 100 (62.5%) had a history of hypertension. Majority of the respondents 54 (54.0%) had been hypertensive for not more than 10 years. Among the respondents, 122 (76.3%) had normal BP at presentation while 38 (23.7%) had elevated BP at presentation. Majority of the respondents took alcohol 93 (58.1%), while 127 (79.4%) of the respondents never smoked tobacco. Most of the respondents 116 (72.5%) had normal weight, while 7 (4.4%) were obese (Table 2).

**TABLE 2:** Lifestyle and health characteristics of the respondents.

Variables	Frequency	Percentage
**History of hypertension (*N* = 160)**		
Yes	100	62.5
No	60	37.5
**Duration of hypertension (*N* = 100)**		
0–10 years	54	54.0
11–20 years	28	28.0
21–30 years	6	6.0
30 years +	12	12.0
**Blood pressure at presentation (*N* = 160)**		
Normal	122	76.3
Elevated	38	23.7
**Alcohol use (*N* = 160)**		
No	67	41.9
Yes	93	58.1
**Tobacco smoking (*N* = 160)**		
Yes	33	20.7
No	127	79.3
**Weight status (*N* = 160)**		
Normal weight	116	72.5
Overweight	37	23.1
Obese	7	4.4

**FIGURE 1:**
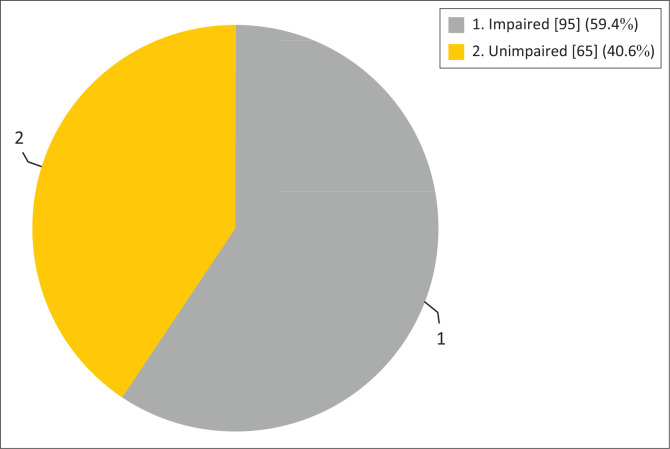
The prevalence of mild cognitive impairment among the respondents in the study.

**FIGURE 2:**
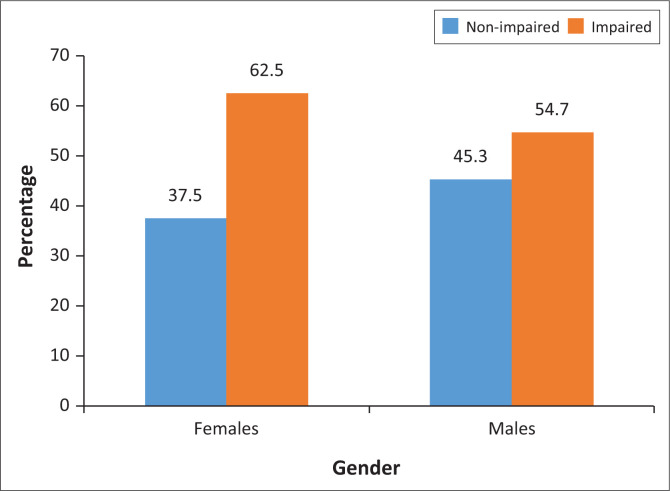
The prevalence of mild cognitive impairment among female and male respondents in the study.

The prevalence of MCI was 59.4% among the respondents (Figure 1). Mild cognitive impairment prevalence was higher among the female respondents than the male (Figure 2).

The highest prevalence of MCI (81.8%) was found among those with no formal education. No formal education was found to be significantly associated with MCI (*p* = 0.006). The following categories had higher prevalence although not statistically significant, age range 75–84 years old 35 (67.3%), female respondents 60 (62.5%), those living in a rural area 41 (70.7%) and widowed respondents 49 (67.1%) (Table 3).

**TABLE 3:** The relationship between sociodemographic characteristics and mild cognitive impairment among the respondents.

Variable	Non-impaired		Impaired		*χ* ^2^	*p*-value
	*n* = 65	%	*n* = 95	%		
**Age (years)**						
65–74	44	44.9	54	55.1	2.100	0.350
75–84	17	32.7	35	67.3	-	-
> 85	4	40.0	6	60.0	-	-
**Gender**						
Female	36	37.5	60	62.5	0.9717	0.324
Male	29	45.3	35	54.7	-	-
**Educational qualification**						
No formal	4	18.2	18	81.8	12.4964	0.006†
Primary	24	33.8	47	66.2	-	-
Secondary	16	50.0	16	50.0	-	-
Tertiary	21	60.0	14	40.0	-	-
**Residence**						
Urban	28	46.7	32	53.3	4.8380	0.089
Semi urban	20	47.6	22	52.4	-	-
Rural	17	29.3	41	70.7	-	-
**Marital status**						
Widowed	24	32.9	49	67.1	3.3415	0.068
Married	41	47.1	46	52.9	-	-

† , Statistically significant.

Among the respondents with history of hypertension, 60 (60.0%) were cognitively impaired. However, this finding was not statistically significant. Those who have been hypertensive for 21–30 years 6 (100.0%) were all cognitively impaired, but this was also not statistically significant. There was no significant relationship between MCI and BP at presentation (*p* = 0.739), although a high number of respondents 74 (59.8%) who had normal BP at presentation, were cognitively impaired (Table 4).

**TABLE 4:** Lifestyle and health characteristics of the respondents and mild cognitive impairment.

Characteristics	Non-impaired		Impaired		*χ* ^2^	*p*-value
	*n* = 65	%	*n* = 95	%		
**History of hypertension**						
Yes	40	40.0	60	60.0	0.0432	0.835
No	25	41.7	35	58.3	-	-
**Duration of hypertension**						
0–10 years	24	44.4	30	55.6	5.159	0.149
11–20 years	10	35.7	18	64.3	-	-
21–30 years	0	0.00	6	100.0	-	-
30 years +	6	50.0	6	50.0	-	-
**Blood pressure at presentation**						
Normal	49	40.2	74	59.8	0.1114	0.739
Elevated	16	43.2	21	56.8	-	-
**Alcohol use**						
Yes	39	41.9	54	58.1	0.1581	0.691
No	26	38.8	41	61.2	-	-
**Tobacco smoking**						
Yes	11	33.3	22	66.7	0.9814	0.322
No	54	42.9	73	57.1	-	-
**Weight status**						
Normal	42	36.2	74	63.8	4.680	0.102
Overweight	18	48.6	19	51.4	-	-
Obese	5	71.4	2	28.6	-	-

MCI, Mild cognitive impairment.

Note: There was no statistically significant association between hypertension and MCI, (two-tailed *p* = 0.149) or between weight status and MCI (two-tailed *p* = 0.102).

The prevalence of MCI was higher (67.7%) among tobacco smokers when compared with those who do not smoke (57.1%), also the prevalence of MCI among the respondents who use alcohol was 61.2% as against 58.1% among those who do not use alcohol. These findings were not found to be statistically significant (Table 4).

Respondents with normal weight 74 (63.8%) had the highest prevalence of MCI. There was no statistically significant relationship between MCI and weight status (*p* = 0.102) (Table 4).

Logistic regression analysis shows the odds of variable, namely education, marital status and weight status that were closest to being significantly associated with MCI when tested at bivariate level. In persons with primary education, the odds of occurrence of MCI are 47% less (OR = 0.53, 95% CI = 0.153–1.819, *p* = 0.311), while in persons with tertiary education, the odds of occurrence of MC1 are 82% less (OR = 0.18, 95% CI = 0.465–0.719, *p* = 0.015). There was a dose-response relationship between education and MCI in this study. This means that as education improves among participants, the likelihood of having MCI was less. Among the married participants, the odds of occurrence of MCI are 19% less (OR = 0.81, 95% CI = 0.394–1.675, *p* = 0.573) (Table 5).

**TABLE 5:** Logistic regression depicting the odds ratios for the occurrence of mild cognitive impairment with education, marital status and weight status.

Variables	Odds ratio	95% Confidence interval (lower – upper)	*p*-value
**Educational qualification**			
No formal education†	1.0	-	-
Primary	0.53	0.153–1.819	0.311
Secondary	0.26	0.069–1.008	0.051
Tertiary	0.18	0.465–0.719	0.015*
**Marital status**			
Widowed†	1.0	-	-
Married	0.81	0.394–1.675	0.573
**Weight status**			
Normal weight†	1.0	-	-
Overweight and/or obese	0.50	0.239–1.058	0.070

† , Reference category.

* *p* < 0.05 (statistically significant).

## Discussion

### Sociodemographic characteristics of the study population

The majority of the study participants were females. This is in keeping with findings in several studies.^8,22,23,24,25^ Ogunniyi et al. in a study carried out in Oyo state, Nigeria, had a female population of 60.7% in their study.^1^ Yusuf et al. in another study carried out in Zaria, Nigeria, had a female population of 60.2%.^26^ Women tend to live longer than men, with the result that there are more older women worldwide than older men.^27^ In 2012, for every 100 women aged 60, there were 84 men.^27^ The proportion of women rises further with age. For every 100 women aged 80 or over worldwide, there are only 61 men.^27^

The study population had a mean age of 72.9 ± 6.4 years. Similar finding was reported in a study carried out by Ogunniyi et al. among older adults.^1^ A systematic review carried out by Adeloye et al. in Nigeria also reported a mean age of 74.4 years.^9^ In this study, majority of the respondents were in the 65–74 years age range. Gureje et al. in a study carried out among older adults in Oyo state Nigeria also reported that the majority of the respondents in their study were in the 65–74 years age range.^21^ Toure et al. in Dakar, Senegal, also reported similar findings.^28^ About a quarter (26.2%) of this study population was still active while most were retired as expected with the study population’s age range.

Among the respondents, there was almost an equal distribution in respondents place of residence as those who reside in the urban area almost equals those residing in rural areas. Place of residence may have a direct impact on older persons because of shrinking social networks, amenities and longer exposure to the biological and psychological conditions in the area of residence.

Most of the respondents in this study had primary school education. This was similar to the findings of Onwuekwe in a study carried out in South-East Nigeria in which the majority of the respondents had primary school education.^3^ Over half of the respondents were married, and those who were not married were either widows or widowers. Widowed individuals have been known to be at an increased risk of psychological morbidity and higher overall mortality compared with married individuals.^29^ All the respondents were Christians; this is not surprising as the study location is made up of mostly Christians.

### Lifestyle and health characteristics of the respondents

Most of the subjects in the study had a history of hypertension with 54.0% having 0–10 years duration of hypertension. This is similar to studies carried out by Ramlall et al. and Kobayashi et al. both in South Africa that had an elderly hypertensive population of 62.8% and 58%, respectively.^30,31^ Nagai et al. in Japan and Alkhunizan et al. in Saudi Arabia got a hypertensive population of 62.8% and 74.3%, respectively.^32,33^ Hypertension is a common disorder among elderly adults and the prevalence tends to rise as the population ages.^34^

More than half of the respondents consume alcohol. This is in line with the findings by Gureje et al. in Ibadan, Nigeria, where 53.8% population consumes alcohol.^21^ Most of the respondents in this study have never smoked, while only a few are currently smoking. This was similar to the study carried out in Tanzania by Putnam et al., which had a 28.5% population that smoked.^35^ Alkhunizan et al. in Saudi Arabia and Fu et al. in China had similar values of 91.8% and 86.5%, respectively, of the respondents who never smoked as was seen in this study.^32,36^ In contrast, a study by Ramlall et al. in South Africa reported that 76.4% of the study population that were current smokers or ever smoked.^30^ This discrepancy in smoking habits may be attributed to socio-cultural and religious differences of the different regions where the studies were conducted. Majority of the respondents had normal weight status; those who were overweight and obese were in the minority. Houle et al. in South Africa similarly found obese subjects to be in the minority and non-obese subjects in the majority in a study among similar population.^37^

### Prevalence of mild cognitive impairment

The prevalence of MCI in this study was high at 59.4%. Most previous studies among older adults in SSA reported lower prevalence rates ranging between 9.0% and 37.9%.^38,39,40,41^ A study carried out by Ogunniyi et al. in Oyo state, southern Nigeria reported MCI prevalence of 18.1%.^1^ Guerchet et al.^38^ found a prevalence of 10.4% in Benin Republic; Clausen et al.^39^ found a prevalence of 9.0% in Botswana; Mbelesso et al.^40^ found a prevalence of 37.9% in Congo, while Tianyi et al.^41^ in a study carried out in Cameroon got a prevalence of 33.3%. Several reasons could be adduced for the high prevalence of MCI in this study. Firstly, in contrast with previous studies that were carried out within the communities, this study was hospital based. Secondly, it is known that older adults commonly present with multi-morbidities,^42^ Thirdly, majority of the respondents in this study had a history of hypertension, used alcohol and had low educational qualification, which are all factors reported to impact on MCI.^25,41,43,44^ Therefore, in this study, there may be a true variation in MCI prevalence because of educational levels, prevalence of behavioural risk factors and management of medical risk factors. Another reason can be the varied MCI diagnostic criteria that were used in the different studies; this could also account for the wide variations in MCI prevalence reported in the different studies.^38,39,40,41^ Over 30 studies on the prevalence of MCI, nearly all from high-income countries in North America, Europe, Australia, Japan and Korea also reported widely varied MCI prevalence that ranged between 3% and 42%.^43^ The prevalence of MCI found in this study was, however, lower than the prevalence found in a study performed in Thailand, which had a prevalence of 71.4%.^23^ Low education and underlying disease associated with MCI were some of the reasons cited for the high prevalence of MCI in the study.

### Prevalence of mild cognitive impairment and association with sociodemographic factors

#### Prevalence of mild cognitive impairment and age

There was a higher prevalence of MCI among respondents who were above 75 years of age, although there was no significant association between the respondents’ age range and MCI. Similarly, in their studies, Gureje et al. in Nigeria,^44^ Tianyi et al. in Cameroon,^41^ Griffiths et al.^23^ in Thailand and Tsoy et al.^43^ in Kazakhstan also found that the prevalence of MCI increased with age despite methodological differences. Advanced age is associated with an increased prevalence of cognitive impairment.^29,30,37^ It has been reported in most studies that age is one of the most important risk factors for cognitive impairment and this was consistently documented in virtually all the aforementioned studies.

#### Prevalence of mild cognitive impairment and gender

Female respondents in this study had a higher prevalence of MCI than their male counterparts; however, this was not statistically significant. The reported prevalence of MCI in females is in keeping with several other results from different studies. Ogunniyi et al.,^1^ Guerchet et al.,^38^ Paddick et al.^22^ and Ramlall et al.^30^ from Nigeria, Benin, Tanzania and South Africa, respectively, all reported similarly higher prevalence of MCI among the female respondents in their studies. It has been postulated that females may be more at risk of developing MCI because of hormonal differences and brain development factors. Oestrogen, progesterone and testosterone have been shown to have neuroprotective effects, with a decrease in these hormones in postmenopausal women possibly accounting for the high prevalence of cognitive impairment among elderly women.^41^ However, other protective factors may exist that are unique to women in developing countries; identification of such factors could be useful in reducing the risk to women in developing countries.^30^ One of such association may be ascribed to the old cultural practice of limited education opportunities for female children in Nigeria. Individuals with limited education have been reported to be more likely to develop MCI.^43^

#### Prevalence of mild cognitive impairment and education

Fewer years of education have been associated with a greater risk of neurocognitive impairment compared with individuals with higher level of education.^43^ In this study, respondents with no formal education had a significantly higher prevalence of cognitive impairment than those with secondary and tertiary levels of education. Extant dementia literature, particularly from the West, and studies from Africa have also demonstrated an association between low educational attainment and increased dementia risk.^23,41,43,45^ Several other studies also reported a decreasing prevalence with increasing educational attainment.^28,31,41,43^ In this study, the respondents with tertiary education were 82% less likely to have MCI. This finding has not been found to be consistent in all studies; Adeloye et al. in a systematic review found a lower prevalence of MCI among those with no formal education.^9^ Other studies have also reported lower prevalence of MCI among study subjects with no formal education.^28,31,43^ A study carried out in Benin, West Africa, did not find any significant association between education and cognitive impairment.^38^

#### Prevalence of mild cognitive impairment and residence

The respondents who stayed in rural areas had a higher prevalence of MCI in this study. Gureje et al. found a greater incidence of cognitive impairment among rural residents, which is similar to the results in this study.^46^ As a result of rural to urban migration of the younger adults, the older adults may be deprived of cognitive stimulating activities derivable from social interaction with the younger population, this may increase their likelihood of developing MCI.^21^ Findings regarding urban and rural residence and prevalence of MCI in the literature are, however, inconsistent. This inconsistency in the prevalence of MCI with respect to the place of residence may be because of the different population densities and definitions of rural in the different studies.

#### Prevalence of mild cognitive impairment and marital status

There was a high prevalence of MCI among widowed respondents in this study. This is consistent with the findings of a systematic review, which reported that the risk of neurocognitive impairment in a Nigerian population is associated with widowhood.^9^ Similar findings had been reported by Guerchet et al.^38^ in Benin and Xu et al.^29^ in China. Unmarried, divorced or widowed persons may have a different lifestyle when compared with those of married people. Xu et al. reported that living without partners may increase the risk of smoking and alcohol consumption, having irregular breakfast and prevent them from undergoing health screening.^29^ A Korean study reported that married adults were generally found to have healthier lifestyle behaviours.^47^ However, higher prevalence of MCI has also been reported among married subjects than unmarried subjects. Toure et al. in Senegal and Griffiths et al. in a study performed in Thailand got a higher prevalence of neurocognitive impairment among married subjects.^23,28^

### Lifestyle and health characteristics of the respondents and mild cognitive impairment

Literature suggests that treatment for hypertension by itself appears to exert a protective effect against MCI, but this study showed that normal BP at presentation and elevated BP at presentation had no impact on the prevalence of MCI. Similarly, no significant association was found among respondents with a history of hypertension and duration of hypertension. The finding of this study is in consonance with some other studies,^23,31,35,38^ which might imply that hypertension on its own does not affect cognition, but might do so in the presence of other comorbid conditions and other lifestyle risk factors.

Pertaining to lifestyle issues, in this study, alcohol use, tobacco smoking and weight status were not found to be significantly associated with MCI prevalence. The literature on this has been mixed with some finding that smoking increased the risk of dementia, especially of the Alzheimer type, and others finding no such link.^44^ A review of literature from Africa also demonstrated limited evidence on the relationship between lifestyle factors such as smoking, alcohol use and cognitive impairment.^45^ Gureje et al. found an increased risk of dementia among older persons reporting a lifetime use of alcohol.^44^ They stated, however, that the link between alcohol consumption and dementia appears not to be a simple one as some studies have suggested that a little alcohol protects against the development of cognitive decline, while excessive consumption increases risk.^44^ Fekadu et al.^11^ in their study, stated that the elderly who use alcohol throughout their lifetime were three times more likely to develop neurocognitive impairment. The advanced reason was that alcohol would prevent the regeneration of neurons and cause deterioration of cognitive ability as well as affect memory formation. Anthropometric markers of malnutrition, such as reduced BMI, have however been associated with the risk of dementia.^45^

### Limitations

This is a cross‑sectional study, thus causality cannot be determined. Some of the data regarding lifestyles and health characteristics of the respondents were obtained by self-report, which might be subject to recall bias. Because cognitive impairment is multifactorial, older persons often have many other risk factors that were not assessed in this study, such as depression, anxiety, atherosclerosis, cardiovascular disease and diabetes mellitus.

### Recommendations

The current knowledge of MCI is limited by inconsistent findings, recognising the earliest stage of cognitive impairment in which the patient has not started showing any functional impairment is therefore very important so that purposeful interventions against the identified risk factors can be instituted. Education was the only modifiable risk factor identified in this study; therefore, advocacy for free compulsory public education up to secondary level and aided education up to tertiary level through innovative programmes and initiatives by the government is recommended.

Further large-scale community-based epidemiological studies assessing the prevalence of MCI and its modifiable risk factors are needed in the pursuit of prevention and early intervention. Screening for MCI and known risk factors should be prioritized at geriatric clinics.

## Conclusion

Mild cognitive impairment is a common problem among older adults in this study. Mild cognitive impairment was found to be significantly associated with individuals with no formal education.
